# Radon (^222^Rn) concentrations in the touristic Jumandy cave in the Amazon region of Ecuador

**DOI:** 10.1093/jrr/rrz064

**Published:** 2019-10-28

**Authors:** Felipe Alejandro García Paz, Yasser Alejandro Gonzalez Romero, Rasa Zalakeviciute

**Affiliations:** 1 Universidad de las Américas, Facultad de Ingeniería y Ciencias Aplicadas, calle José Queri y Av. de los Granados/Bloque 4, Quito – EC 170125, Ecuador; 2 Grupo de Biodiversidad Medio Ambiente y Salud (BIOMAS), Universidad de Las Americas, calleJosé Queri y Av. de los Granados/Bloque 7, Quito – EC 170125, Ecuador

**Keywords:** natural radiation, radon exposure, cave monitoring, health risk, effective dose

## Abstract

This work consists of the detection and quantification of the concentration levels of radioactive gas radon-222 (^222^Rn) of natural origin, as well as the determination of the critical points and the estimation of the effective dose absorbed by the tourists and guides inside the Jumandy cavern in Napo, Ecuador. According to the feasibility map of uranium of Ecuador, the study area is located in one of the top-priority areas for obtaining uranium, suggesting possible radioactivity in this unstudied region. The measurements were carried out from July to October of 2017, in three different monitoring points inside the cavern. The average radon concentrations measured in the cavern exceeded the maximum recommended environmental level by a factor of 28, and the effective dose absorbed by the guides exceeded the recommended maximum by a f actor of 10. Meteorological parameters such as temperature and relative humidity have an impact on the ^222^Rn concentrations in different parts of the cave.

## INTRODUCTION

Radioactivity is a physical phenomenon, where the nucleus of an atom has many or very few neutrons, causing the atom to become unstable and, in its search for stability, generate or emit radiation. Naturally occurring radioactive materials (NORM) are present in many minerals and raw materials of the planet and are well studied [31]. Among them, radon-222 (^222^Rn) is a noble radioactive gas that results from radium-226 (Ra^226^) decay and has a half-life of 3.8 days [24]. In the chain of radioactive decay of uranium (^238^U) every radioisotope eventually decays into a stable element such as lead (^206^Pb). Radon is naturally present at low concentrations in the air we breathe, but is difficult to detect because it is colorless, odorless and tasteless. Radon is highly soluble in water and can be present in soil. Previous studies have identified water as a potentially significant source of indoor ^222^Rn [22]. Recent studies even correlate soil radon gas anomalies with seismic activity [32]. Moreover, indoor radon studies show the importance of environmental parameters, such as positive correlation with indoor humidity and outdoor temperature, and negative correlation with barometric pressure and wind speed for well ventilated spaces, whereas for unventilated spaces it is positively correlated with outdoor hourly rainfall [38]. This gas is more concentrated in spaces with little ventilation and near the ground, causing health concerns [26].

When inhaled, radon adheres to the lung tissues where the bronchopulmonary cells are irradiated (WHO [34], [35]). The ionizing particles from the radioactive decay of ^222^Rn (^218^Po, ^214^Po) interact with the lung tissues, causing alterations in the DNA of the lung cells [26]. Therefore, it is the main cause of lung cancer deaths for non-smokers worldwide, and is considered a human carcinogen by the Oncological Research department of the World Health Organization [23]. In addition, the alteration of the DNA of the lung cells, as a consequence of exposure to radon gas, can occur at any concentration [7].

**Fig. 1:**
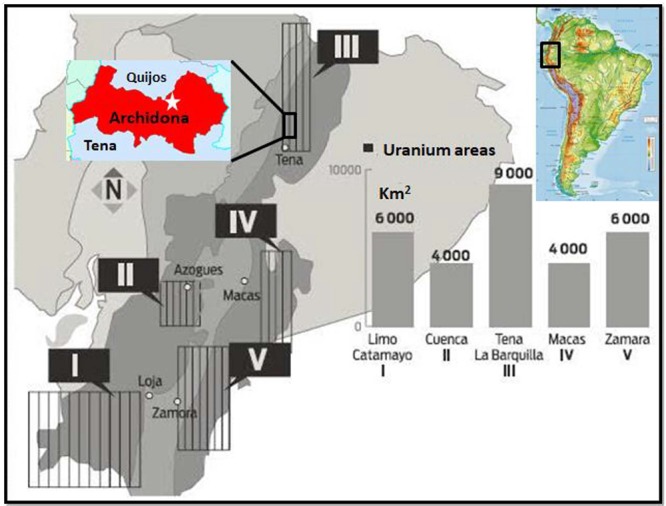
Uraniferous feasibility areas of Ecuador INIGEMM [17]. Geological maps of the study area (upper left) located in the uraniferous zone of the Ecuadorian Andes, South America (upper right). Maps modified from INIGEMM, [17]; and [11]. The study area is marked by a star. The grey shaded areas indicate priority uraniferous zones (darker grey—higher prioriy to lighter grey—lower priority areas.)

According to the geological map of Ecuador, the Archidona caves of Napo province are located in an area of igneous geology. This type of rock has in its structure the presence of uranium and thorium, the elements that precede the generation of radon [4]. The caves are ideal settings for the presence of natural-origin radioactive gases such as radon, which should be considered, following the principles for radiological protection [16], since it is a tourist destination. Based on the lack of studies on ^222^Rn concentration measurements in Ecuador, and in general in South America, we highlight the importance of this study for public health interest. There are no records of previous studies on ^222^Rn concentration measurements in caves in the Amazon region of Ecuador or in the Andes mountains (see darker grey shaded areas in Fig. 1).

## MATERIALS AND METHODS

The study was conducted in the caves of Jumandy, about 15 km north of Tena, and 2 km north of Archidona, Napo, in the Amazon region of Ecuador (see Fig. 1). The study area is located at an elevation of 635 m.a.s.l., (meters above sea level) exposed to average relative humidity of 75% and average atmospheric pressure of 903 hPa. According to the uraniferous feasibility map of Ecuador, Tena is located in an area where radiometric anomalies exist (see Fig. 1), and for this reason place was established as an ideal area to study ^222^Rn concentrations. The characteristics, such as dimensions and shape of the cavern, as well as a daily flow of people who are exposed to ^222^Rn, were considered.

The monitoring was carried out at 3 different points in the cavern during the dry season (July—August) and wet season (September—October) of 2017, by using diffusion cameras as an active detection instrument. The monitoring points were determined according to the tourists flow in the different areas of the cave. The equipment to detect the ^222^Rn was placed 50 cm away from the ground and 25 cm away from the wall. The monitoring of the physical parameters was performed by measuring the relative humidity, temperature, pressure and wind speed. All these parameters allowed us to analyze a correlation between the different sampling points and the radon concentrations. The monitoring was carried out in the morning and in the afternoon, when there is the greatest number of tourists.

A survey of the geological characteristics of the region was carried out, as the ^222^Rn varies in different types of soil or minerals. The National Institute of Metallurgical Mining and Geological Research of Ecuador, generated a geological map in 1986 showing that the study area is located between Tena and Napo Formations (Fig. 1). The Tena formation is located between the cities of Tena and Archidona. It is formed by conglomerate calcareous sands, medium and fine sandstones interspersed with siltstones and arcillolites. It is located in a geological time scale called the Cretaceous Paleocene. The Napo formation is located in the elevated area of ​​the Napo River and its lithology is divided into three parts of Lower Napo, Medium Napo and Upper Napo. Glauconitic sandstones and sandy shales as well as clastic texture limestones are found in the Lower Napo section.

To carry out the mineralogical analysis, the samples were scraped with a tungsten carbonate skimmer. Once the samples were scraped it was necessary to apply a 10% hydrochloric acid solution [3]. Finally, the mineralogical properties of every sample were determined using a magnet and a magnifying glass.

To calculate the estimated effective dose received by the guides and tourists inside the caves, concentration of ^222^Rn, dose-conversion factors and alpha radiation by the decay products of ^222^Rn (^214^Po and ^218^Po) were taken into account. In the uranium-238 (^238^U) radioactive decay chain, radon-222 (^222^Rn) has a half-life of 3.82 days, after this time unstable which ^222^Rn will decay into ^218^Po and ^214^Pb [24].

The estimated effective dose was determined according to the data obtained at the three points of the touristic cave applying the commonly used equation of Papachristodoulou [29]. This equation has been previously used in other studies inside caverns.

The measurement points were determined by the stops made by the guides inside the cave to explain and clarify specific details to the tourists inside the cave.

${E}_{\mathrm{Rn}}={C}_{\mathrm{Rn}}\times F\times t\times d\times u$ (1)

where:

*E*_Rn_ = effective dose (mSv/year).

*C*_Rn_ = radon concentration inside the cave (Bq/m^3^).

*F* = average of the equilibrium factor between radon and decay products. F = 0.57.

*t* = time inside the cave (h/year).

*d* = dose conversion factor. 1.4 for workers (mSv/mJ h/m^3^).

*u* = constant- unit factor of 5.6 x10^−6^ [(mJ/m^3^)/Bq/m^3^].

**Fig. 2:**
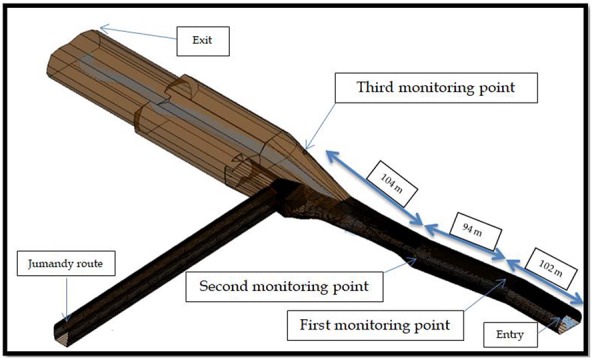
Dimensions and points of interest inside the Jumandy cave in Archidona, Ecuador.

The guided tours are organized in groups of up to 10 people. Each tour has a duration of ~1 h to complete the distance of 900 m within the cavern. The entrance is in the east and the exit is in the north (see Fig. 2). During the tour, the guide in charge stops the group at three points inside the cave for explanations.

Within the experimental methodology, models were applied allowing the correlation analysis of data obtained during the measurements. The data obtained were processed in an integrated development environment with the help of Rstudio software using ‘*Psych*’ and ‘*Pairs Panels*’.

During the 4-months field study we registered that in the touristic Jumandy cave, the ^222^Rn concentrations range between 156 Bq/m^3^ and 4100 Bq/m^3^. These levels exceed the highest permissible level (148 Bq/m^3^) by a factor of 28 [36]. Low concentrations were registered during the dry season (July–August) and high concentrations during the wet season (October). The peak concentrations of this study compare well with the radiation levels in other countries ([1], [2], [6], [8]). Whereas most of the studies are reported from Europe and Australia, at this time there is a very limited number of research studies on ^222^Rn levels in South America.

### Point 1—cave entrance

At the first monitoring point an average ^222^Rn concentration of 1381 Bq/m^3^ was obtained (see Table 1). The minimum concentration value of ^222^Rn was 421 Bq/m^3^ in August and the maximum was 2116 Bq/m^3^ in October.

**Table 1:** Dates and parameters measured in the: 1, first monitoring point—cave entrance; 2, second monitoring point—middle cave; and 3, third monitoring point—deep cave

Date	Radon concentration Bq/m			Temperature			Reative humidity			Atmospheric pressure			Wind speed		
	Bq/m^3^			^o^C			(%)			(hPa)			(m/s)		
	1	2	3	1	2	3	1	2	3	1	2	3	1	2	3
18/7/2017	1236			23.4			84			933			0.3		
19/7/2017	1395	156		24	23.5		85	79.8		937	938		0.32	0.4	
20/7/2017		167	174		24	24		83.5	83.5		940.2	938		0.43	0.43
21/7/2017			167			30.3			71.6			937			0.54
26/8/2017	476			24.2			83.4			939.03			0.03		
27/8/2017	421	637		25.9	26.2		84.4	82.1		941	943.34		0.46	0.35	
28/8/2017		703	811		26.2	24.5		77.2	84		941	943.3		0.51	0.63
29/8/2017			761			27.9			68.6			942			0.96
28/9/2017	1719			27.3			74.6			939.62			0.19		
29/9/2017	1662	1380		26.3	26.7		76.1	78.2		941.23	934.88		0.19	0.22	
30/9/2017		1388	777		23.1	26.6		85.3	78		935	930.95		0.64	0.48
1/10/2017			803			26.8			79			933.4			0.22
26/10/2017	2025			25.8			76.6			937			0.38		
27/10/2017	2116	3789		30.1	24.2		63.1	86.6		938	937		0.77	0.33	
28/10/2017		1144	4100		23.9	24.2		86.7	86.6		938	942		0.08	0.33
29/10/2017			3087			22			85.6			941			0.28
Average	1381.3	1170.5	1335.0	25.9	24.7	25.8	78.4	82.4	79.6	938.2	938.4	938.5	0.3	0.4	0.5

**Fig. 3:**
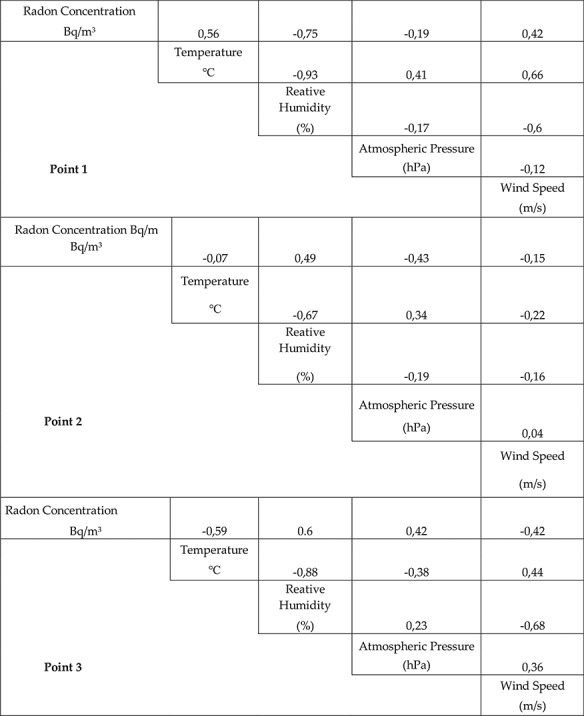
Correlations between the concentration of ^222^Rn and temperature, relative humidity, pressure and wind speed at all three monitoring points.

Fig. 3 shows that there is a positive correlation between the concentration of ^222^Rn and temperature (*r* = 0.56), and the concentration and wind speed (*r* = 0.42). This could be a result of the position of this point at the entrance of the cave (see Fig. 2), where the variations in temperature and wind speed would have a direct positive effect on the transport of the radiation. We note that wind flow inside of the cave follows the length of the cave towards the exit (from Point 3 to Point 1, see Fig. 2). Therefore, the elevated average concentrations of radon might be a result of an accumulation of radiation at this point.

The correlation between ^222^Rn concentration and relative humidity was significant negative (*r* = −0.75), and correlation between ^222^Rn concentrations and pressure was weak negative (*r* = −0.19) (see Fig. 3). This might indicate that during the increased relative humidity (rain) events, the atmospheric turbulence decreases and inhibits ventilation into the cave, reducing the transport of radiation. When there is an efficient air movement inside caves, the ^222^Rn concentration tends to decrease [27].

### Point 2—middle cave

An average ^222^Rn concentration of 1170 Bq/m^3^ was obtained at the second point, located in the middle of the touristic route (see Table 1 and Fig. 2). The minimum concentration value of ^222^Rn at the second point was 156 Bq/m^3^ in July and the maximum was 3789 Bq/m^3^ in October (Table 1). This point displayed a higher variation than the first point. The ^222^Rn concentrations at this point compare well with the data obtained in similar research done in Firepole cave, The Bahamas [6]. However in our study, the ^222^Rn concentration is slightly higher.

We show that there is a positive correlation (*r* = 0.49) between the concentration of ^222^Rn and the relative humidity at this point, whereas the parameters such as temperature, pressure and wind speed showed a weak or insignificant negative relationship with the concentration levels of ^222^Rn (see Fig. 3). In this case, the wind speed is not a parameter that determines the ^222^Rn concentrations (see Fig. 3). This could be explained by the fact that inside the cave, just behind the second measurement point, there is a natural waterfall formed by groundwater. This could cause the variability of wind speed data because when the groundwater flow increases, it might affect the variability in wind speed, being far from the entrance of the cave. On the other hand, inside the cave, the relative humidity has a positive effect on ^222^Rn concentration, explained by the drop in temperatures and a resulting increase in emissions of the radiation gas, also registered in previous studies [8]. This suggests that deeper points of the cave will contain higher concentrations of radon.

### Point 3—deep cave

The average ^222^Rn concentration at the third point of the Jumandy cave was 1335 Bq/m^3^ (see Table 4). The minimum ^222^Rn concentration was 167 Bq/m^3^ in July and the maximum value was 4100 Bq/m^3^ in October. It was determined that the maximum concentration values of ^222^Rn obtained during the study were registered at this deepest point of the cave. However, the average ^222^Rn concentrations were exceeded by 46.25 Bq/m^3^ at the first monitoring point.

Similar to the second monitoring point, the decrease in temperature and increase in relative humidity would result in increasing ^222^Rn concentrations, whereas the increase in wind speed would increase the ventilation out of the cave and transport the radiation to the other parts of the cave (Fig. 3). In this case these effects were more significant.

### Meteorology and radiation

**Fig. 4:**
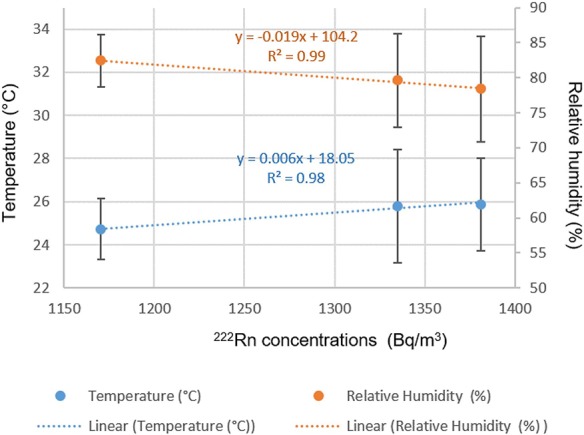
Linear correlation analyses between average ^222^Rn concentration and temperature and relative humidity.

As Gallego *et al*. [10] explained in a similar study in Castañar, Spain, temperature variations caused by seasonal changes would affect relative humidity and therefore ^222^Rn concentrations. In addition, the increase in emissions of the radiation gas during the cold season was previously discussed [8]. Although temperature and relative humidity variations are small in the Amazon region of Ecuador, the precipitation accumulation would be the defining parameter in tropical season variation (wet and dry season). However, even small changes in temperature and relative humidity might impact the average levels of ^222^Rn. In Fig. 4, a strong positive correlation between average temperatures and average ^222^Rn concentrations at different measurement points is seen. This suggests that even a slight increase in temperature variation in a tropical climate zone might increase overall levels of radiation in natural caves.

**Fig. 5:**
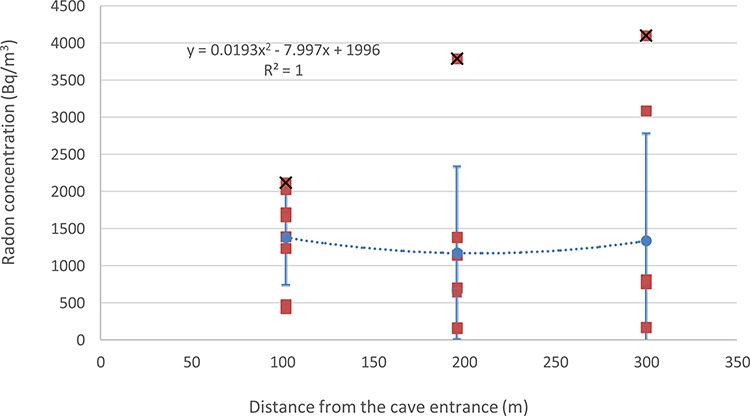
Correlation analysis of the overall average (blue markers) and all 24-hour measured (orange markers) ^222^Rn concentrations in the three measurement points of the Jumandy touristic cave depending on their distance away from the cave entrance. The peak concentrations are marked with a black cross.

However, the meteorological parameters play a different role depending on the distance from the cave entrance, showing a strong negative effect of peak temperatures in the deeper end of a cave. We present a summary in Fig. 5 that shows the variation in the peak concentrations of ^222^Rn inside the cave depending on the point’s distance from the entrance. This indicates that the peak concentrations are influenced by the lack of ventilation. The results of our study confirm the findings in karstic caves in Venezuela, where the distance from the entrance caused changes in the concentrations of ^222^Rn [2].

**Figure A.1:**
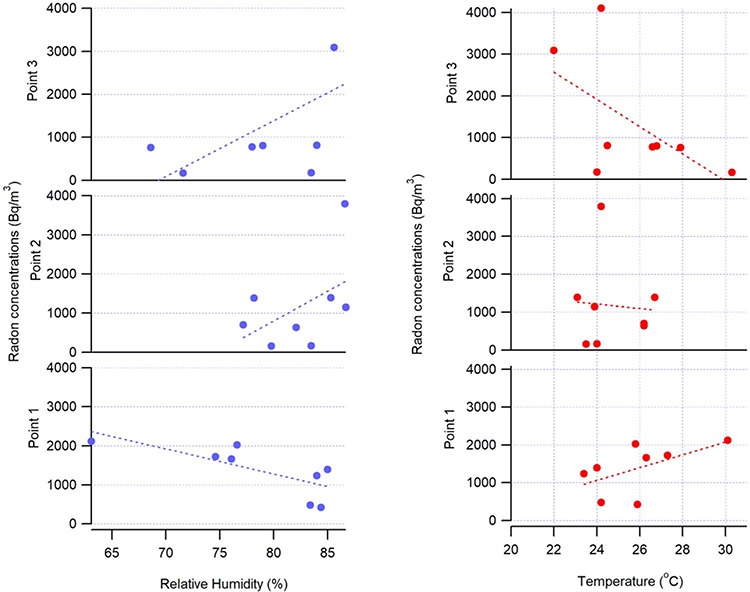
Correlation analysis of ^222^Rn concentrations and relative humidity (left panel), and temperature (right panel) in the three measurement points of the Jumandy touristic cave.

In addition, our data shows that Point 1 (closest to the entrance) shows an opposite effect if compared with the other two points located deeper in the cave (see Fig. A.1 and Fig. 5). As previously suggested by other studies [38], higher ventilation rates cause not only a positive correlation between temperature and a negative correlation between relative humidity and ^222^Rn concentrations, but also lower overall levels of this gas. Meanwhile the effect of temperature becomes increasingly more negative and the effect of relative humidity increasingly more positive, with distance away from the entrance. In the humid tropical climate increasing relative humidity often implies precipitation events and a drop in temperatures. Another observation can be made that the variation in Rn concentration is the smallest near the entrance, while the further we get away from the entrance, the peak concentrations appear due to increased relative humidity and lower temperatures.

### Mineralogical analysis

The mineralogical characterization of four samples removed from the Jumandy touristic cave corresponds to bituminous, conglomerate and calcareous sandstones. According to the Paleontological Research Institute [28] the sandstones possess quantities of ^238^U, ^235^U, ^40^K, ^232^Th and ^226^Ra. Depending on the type of sandstone obtained, the levels for ^232^Th can range from 0.7 ppm to 227 ppm and for ^238^U from 0.1 ppm to 62 ppm [25]. This confirms that the source of the radiation levels found in the present study is due to the natural rock formations within the cave (Fig. 1). As the mineralogical properties present in Napo and Tena formations have high ^222^Rn concentrations, this suggests the presence of ^222^Rn in all the countries within the Andean range.

### Effective dose

Finally, the estimate of the effective annual dose received considered: (i) effective dose according to the levels of concentration and period of time spent at each monitoring point for tourists and guides; and (ii) effective dose according to the levels of concentration and period of time spent at each monitoring point, taking into account the average concentration of the three monitoring points for guides. For the first estimate, the time in minutes that tourists and guides stayed at each monitoring point was taken into account and the concentration of ^222^Rn found at each point was averaged.

- Point 1: 1,381.25 Bq/m3 × 0.57 × 0.083 h/year × 1.4x5.6× 10-6 mJ/m3/Bq/m3 = 5.12x10-4 mSv/year
- Point 2: 1,170.5 Bq/m3 × 0.57 × 0.05 h/year × 1.4x5.6 × 10-6 mJ/m3/Bq/m3 = 2.61x10-4 mSv/year
- Point 3: 1,335 Bq/m3 × 0.57 × 0.166 h/year × 1.4x5.6 × 10-6 mJ/m3/Bq/m3 = 9.90x10-4 mSv/year

For the second estimate, the average concentration at the three monitoring points was considered in all the measurements. Also, a 35 hours per week working period for guides was taken into account.

- Points 1-3: 1,295.58 Bq/m^3^×0.57×1680 h/year ×1,4×5.6 ×10^−6^ mJ/m^3^/Bq/m^3^ = 9.72 mSv/year

Both ^222^Rn concentrations and the estimated effective dose received exceed the maximum permitted limits established by the Principles of Radiological Protection [21]. The estimated effective dose received by guides in Jumandy cave exceed healthy limits by a factor of 10 (International Atomic Energy Agency [18], [19], [20]) for personnel exposure: 1 mSv/year), and by a factor of 10 the effective dose found in a similar study in Santana Cave, Brazil [1]. However, we note that most of the reported research studying natural formation caves is performed in non-touristic areas. The findings of this study indicate a real concern for the radiation exposure to the tourists and, especially, to the communities that manage the activities. This is of particular concern to the developing countries that benefit from tourism. Thus, the guide’s exposure has to be limited to 7 h per month so as not to exceed 1 mSv/year.

The findings of this study raise serious concerns due to a higher than background level of exposure to radon gas in this area. A number of studies, performed in other regions where annual effective doses are multiple times higher than the recommended levels, suggest radiation-related health effects [15]. For instance, in Poços de Caldas and Guarapari, Brazil, elevated standardized mortality due to cancer was registered [33]; in Ramsar, Iran (1.6–42 mSv/year), chromosomal aberrations have been recorded ([12]; [13]); in Yangjiang, China (1.98–3.1 mSv/year), non-cancer mortality, including cerebrovascular diseases, tuberculosis, viral infections and diseases of the digestive system (in particular chronic liver disease) were shown [39], etc. Some other regions also demonstrate no biological effects, suggesting adaptive responses to elevated levels of radioactivity. This study could be a valuable precursor to an interesting further study focused on health effects in the area.

## CONCLUSIONS

To the best of our knowledge, this is the first radioactivity study performed in the Andean Mountains, and one of a very few in Latin America. Upon the monitoring of the concentrations of ^222^Rn during the 4 months study, through active detection methods, it was determined that the highest average concentration of ^222^Rn is found in the first monitoring point at the cave entrance (1381 Bq/m^3^) due to the ventilation effect. The wind could be responsible for the transport of ^222^Rn from the inside of the cave. Meanwhile, the highest concentration peaks of ^222^Rn correspond to the monitoring point deep in the cave (4100 Bq/m^3^) during the wet season, due to a drop in temperatures. Our results compare well with other studies done in the world and a few limited Latin American countries. Although studies in the Andean region are absent, our findings suggest the presence of elevated levels of radon in the whole cordillera, implying health concerns for the sierra population of South America. The results of our study show the necessity for further research in this region.

The estimates of the effective annual dose received for guides at the touristic Jumandy caves in the Ecuadorian Amazon region exceeded the maximum permissible limit of 1 mSv/year. The estimated dose received by guides was close to 10 times higher at 9.72 mSv/year, and although it is not the highest in the world, it is higher than the levels reported in another Latin American study. The present research shows that possible health risks associated with ^222^Rn concentrations inside the touristic caves must be considered in order to create national and even regional legislation to protect citizens exposed to high ^222^Rn concentrations.

Finally, the geological analysis of cave samples concurs with the geological characteristics of the Napo Formation and explains the elevated levels of radiation. This suggests that in other parts of the Amazon and Andean border elevated ^222^Rn concentration levels would also be found, giving a good basis for future health impact studies.
